# S100A7 (Psoriasin), highly expressed in Ductal Carcinoma *In Situ *(DCIS), is regulated by IFN-gamma in mammary epithelial cells

**DOI:** 10.1186/1471-2407-7-205

**Published:** 2007-11-06

**Authors:** Stina Petersson, Anna Bylander, Maria Yhr, Charlotta Enerbäck

**Affiliations:** 1 Department of Clinical Genetics, Sahlgrenska University Hospital, SE-413 45 Göteborg, Sweden; 2 Department of Dermatology, Sahlgrenska University Hospital, SE-413 45 Göteborg, Sweden

## Abstract

**Background:**

The aim of the present work was to explore signal transduction pathways used in the regulation of S100A7 (psoriasin). Members of the S100 gene family participate in many important cellular functions. Psoriasin, S100A8 (calgranulin A) and S100A9 (calgranulin B) are expressed in ductal carcinoma *in situ *(DCIS), as well as in the hyperproliferative skin disease, psoriasis. In the latter condition, a disturbance in the STAT pathway has recently been reported. This pathway is implicated in the regulation of IFN-gamma, widely recognized as a key cytokine in psoriasis. IFN-gamma also exerts anti-tumor action in a number of tumor cell types, including breast cancer. We therefore examined the effect of IFN-gamma and STAT-signaling on the psoriasin expression.

**Methods:**

We established a TAC2 mouse mammary epithelial cell line with tetracycline-inducible psoriasin expression (Tet-Off). Viability in cell culture was estimated using MTS assay. Protein and gene expression were evaluated by Western blotting and quantitative real-time PCR. Statistical analyses were assessed using a one-tailed, paired t-test.

**Results:**

We report the downregulation of psoriasin by IFN-gamma in the MDA-MB-468 breast cancer cell line, as well as the downregulation of psoriasin induced by anoikis in cell lines derived from different epithelial tissues. In contrast, IFN-gamma had no suppressive effect on calgranulin A or calgranulin B. IFN-gamma is an important activator of the STAT1 pathway and we confirmed an active signaling pathway in the cell lines that responded to IFN-gamma treatment. In contrast, in the SUM190 breast carcinoma cell line, IFN-gamma did not suppress the expression of endogenous psoriasin. Moreover, a reduced phosphorylation of the STAT1 protein was observed. We showed that IFN-gamma treatment and the inhibition of the transcription factor NFkappaB had a synergistic effect on psoriasin levels. Finally, in TAC2 cells with tetracycline-induced psoriasin expression, we observed the increased viability of psoriasin-expressing cells after IFN-gamma treatment.

**Conclusion:**

Our data support the possibility that psoriasin expression is transcriptionally suppressed by IFN-gamma and that this effect is likely to be mediated by the activation of the STAT1 signaling pathway. The increased viability of psoriasin-expressing cells after IFN-gamma exposure suggests that psoriasin expression leads to the development of an apoptosis-resistant phenotype.

## Background

S100 proteins are characterised by calcium-binding domains and they are involved in many cellular processes such as proliferation, differentiation and cell shape [1]. Three proteins in this family, predominantly psoriasin but also calgranulin A and calgranulin B, have been shown to be highly expressed in DCIS [2-4]. Both we and others have also identified psoriasin as being differentially expressed between DCIS and invasive breast carcinomas and have found a correlation to poor prognosis, suggesting its potential involvement in tumour progression [3-6]. Psoriasin protein was detected in the cytoplasm and the nucleus, but it was also found to be secreted [2,3]. In MCF10A human immortalised mammary epithelial cells, the expression of psoriasin is very low in exponentially growing cells, but it is significantly upregulated by growth factor deprivation, loss of attachment to the extracellular matrix (anoikis), and confluent conditions [3]. Moreover, we showed that psoriasin was induced by reactive oxygen species (ROS) [7]. Since all these conditions influence cell proliferation and survival, psoriasin may play a role in the regulation of these pathways. Consequently, psoriasin expression was shown to correlate to increased survival and NFκB signaling [7,8]. Moreover, psoriasin was found to interact with Jun activating binding protein1 (Jab1), which is involved in multiple signal transduction pathways, including the regulation of JUN/AP1 transcription factors [6]. The over expression of psoriasin in MDA-MB-231 breast cancer cells was shown to increase nuclear Jab1 activity and enhance tumorigenesis *in vivo *in nude mice [6]. We recently showed that the downregulation of endogenous psoriasin expression in the MDA-MB-468 cell line by short hairpin RNAs increased cell migration and invasion without influencing cell proliferation and survival *in vitro *but inhibited tumour growth *in vivo*. In accordance with these findings, we showed an upregulation of matrix metalloproteinase 13 (MMP13) and a downregulation of vascular endothelial growth factor (VEGF) in cells with reduced psoriasin levels [9].

There is evidence that psoriasin can function as a chemotactic factor for CD4+ lymphocytes in the skin and it has recently been implicated in the anti-bacterial defence mechanism of the skin [10,11]. The high expression of psoriasin and calgranulin B was initially observed in *psoriasis *[12,13], which is characterised by the hyperproliferation of keratinocytes, the infiltration of activated T lymphocytes and vascular neogenesis [14], features it shares with neoplastic tissues. Psoriasis is a T-cell-mediated disorder and the production of proinflammatory cytokines like Interferon-gamma (IFN-gamma), Interleukin-1 and Interleukin-12 plays an important role in the psoriatic phenotype. IFN-gamma is one of the key cytokines in psoriasis and is present at high levels in the psoriatic lesion. Its involvement in the pathogenesis of psoriasis is suggested by the finding that uninvolved psoriatic skin develops psoriasis-like lesions at the site of IFN-gamma injection [15].

IFN-gamma has also demonstrated anti-tumor action in diverse tumor cell types [16]. IFN-gamma was shown to inhibit the cell growth of mammary carcinoma cells and also to reduce angiogenesis [17,18]. The characterization of genes that are regulated by IFN-gamma may lead to a better understanding of the anti-tumoral mechanisms. The purpose of this study was therefore to examine the expression of the S100 proteins psoriasin, calgranulin A and calgranulin B in mammary epithelial cells and keratinocytes *in vitro *after IFN-gamma treatment, with the aim of elucidating signal transduction pathways relevant for both DCIS and psoriasis. We report the suppression of psoriasin by IFN-gamma in mammary epithelial cells and in keratinocytes by the activation of the signal transducers and activators of transcription 1 (STAT1) signaling pathway.

## Methods

### Cell lines and culture condition

The primary human keratinocyte cell line, HEKn, was cultured in Epilife medium, supplemented with 1% EDGS and 0. 1% calcium chloride (CaCl_2_) (Cascade Biologics™, Portland, OR, USA). The MDA-MB-468 (mammary breast carcinoma) and MCF10A (immortalised normal breast epithelium) cell lines were obtained from American Type Culture Collection and were cultivated in 10% foetal clone III in McCoy's 5A medium (Gibco, Invitrogen Corporation, UK) and in DMEM/F12 medium (PAA Laboratories GmbH, Pasching, Austria), supplemented with 20 ng/ml epidermal growth factor, 100 ng/ml cholera toxin, 0,01 mg/ml insulin and 500 ng/ml hydrocortisone. The SUM190 cell line (Asterand, Detroit, MI) was cultured in Mammary Epithelial Cell Medium MEGM BulletKit^® ^(cc-3150) (Cambrex Bio Science Walkersville, Inc.). TAC2 (mammary mouse epithelial cells), kindly provided by Professor R. Montesano, was cultured in 10% FBS in DMEM/high glucose medium (Life Technologies, Inc.), supplemented with 20 ng/ml tetracycline, 100 ng/ml hygromycin and 250 μg/ml neomycin. For suspension cultures (anoikis), cells were plated into poly-2-hydroxy-ethylmethacrylate (polyHEMA) (Sigma Aldrich, Sweden) coated (10 mg/cm^2 ^in 95% ethanol) Petri dishes. To investigate the regulation of endogenous psoriasin expression in the MDA-MB-468 cell line, SUM190 cell line and psoriasin induced by anoikis in HEKn and MCF10A, IFN-gamma (Sigma-Aldrich, Sweden) was added to the medium at a concentration of 250–2000 u/ml medium. To examine the effect of the NFκB pathway, MCF10A cells were plated in polyHEMA-coated Petri dishes and infected with 1 μl Green Fluorescent Protein (GFP) or phosphorylation-defective dnIkkβ adenovirus. Adenovirus generation were as previously described [7]. After the indicated time, cells were harvested and immediately frozen in liquid nitrogen. In experiments performed using hydrogen peroxide (H_2_O_2_) (Sigma Aldrich, Sweden), cells were plated and treated the next day with 75 μM H_2_O_2 _for one hour. Cells were harvested after 48 h in regular medium.

### Establishment of tetracycline-inducible psoriasin expression in a TAC2 mouse mammary epithelial cell line

TAC2 cells were kindly provided by Professor R Montesano. We established a cell line expressing the Tet activator (tTA) by transfecting TAC2 cells with the ptTA-IRES-Neo (tTA, tet activator; IRES, internal ribosomal entry; neo, G418 resistance gene) plasmid, selected for neomycin-resistant clones. Next, we subcloned the psoriasin cDNA into pBI-EGFP2. Cells with a high expression of tTA were transfected with the pBI-EGFP-psoriasin plasmid and clones with low background expression and homogeneous psoriasin induction were selected.

### Western blotting

Protein concentrations were determined using the Bio-Rad protein assay (Bio-Rad, Hercules, CA, USA) and a specific amount of protein lysate was run on a Nu PAGE™ 4–12% Bis-Tris Gel (Invitrogen™, Sweden) for one hour, together with 10 μl of MultiMark^® ^(Invitrogen™, Sweden). Proteins were then transferred by blotting to a 140 μm nitrocellulose blotting membrane at 30 V for 1.5 hours in 1× NuPAGE™ transfer buffer (Invitrogen™, Sweden). The membrane was then blocked in 5% skimmed milk powder in Tris-buffered saline-0.05% Tween (1× TBST-buffer) over night. Western blot analyses of cell lysate were produced with anti-psoriasin (mouse-Ab) (Imgenex, San Diego, CA, USA), anti-tubulin β (mouse-Ab) (Neomarkers, Freemont, CA, USA), anti-Stat1α (mouse-Ab), anti-p-Stat1 (rabbit-Ab), anti-calgranulin A (mouse-Ab), anti-Caspase-3 (mouse-Ab) and anti-GAPDH (rabbit-Ab) (Santa Cruz Biotechnology, USA). Moreover, anti-calgranulin B (mouse-Ab) (Dianova GmbH, Hamburg, Germany) and anti-IκB-α (mouse-Ab) (Cell Signaling Technology, USA) were used. Horseradish-peroxidase-conjugated AffiniPure anti-mouse or anti-rabbit (Jackson ImmunoResearch, West Grove, PA, USA) served as secondary antibodies, followed by incubation in SuperSignal^® ^Stable Peroxide Solution together with SuperSignal^® ^West Pico Luminol/Enhancer Solution (Pierce, Rockford, IL, USA), according to the manufacturer's description. The chemiluminescence signal was registered with a FluorChem 8000 camera (Alpha Innotech, San Leandro, CA, USA).

### RNA extraction, cDNA synthesis and quantitative real-time PCR

Total RNA was prepared from cultured cells using an RNeasy^® ^Mini Kit (Qiagen, Maryland, USA), according to the manufacturer's protocol. cDNA was synthesised using SuperScript™II RNase H^- ^Reverse Transcriptase (Invitrogen™, Sweden), as described by the manufacturer. The relative expression of psoriasin was analysed using real-time PCR and the LightCycler instrument (Roche Applied Science, Sweden), together with the LightCycler FastStart DNA Master SYBR Green I kit (Roche Applied Science, Sweden), according to the manufacturer's instructions. Primers for psoriasin [2] and β-actin [19] were as previously described. Two μl of cDNA were added to 18 μl of PCR Master Mixture (including 3 mM of MgCl_2_, 0.5 mM of primers (psoriasin and β-actin) and 2 μl of LightCycler™Fast Start Master SYBR Green). The protocol consisted of a 10-min denaturation step at 95°C and 40–45 cycles of amplification at 95°C for 10 seconds, 58°C (psoriasin) and 60°C (β-actin) for five seconds and 72°C for eight seconds (β-actin) and 10 seconds (psoriasin) respectively. After amplification, melting curves were obtained to verify the specificity of the amplification reaction and the products were also run on a 2% agarose gel. Quantitative analysis was performed with the LightCycler software version 3.5 (Roche Applied Science, Sweden), which estimates the crossing point (CP) for each sample. The CP values determined for psoriasin were normalised to β-actin (endogenous control).

### MTS assay

Viability in cell culture after IFN-gamma exposure was estimated using a CellTiter 96^® ^AQ_ueous _One Solution Cell proliferation assay (MTS) (Promega, Madison, WI, USA), according to the manufacturer's manual. The absorbance at 490 nm was recorded using the Mithras LB940 Instrument (Berthold Technologies).

### Statistical analysis

To study the association between psoriasin expression and viability, psoriasin-expressing cells were compared with control cells using a one-tailed paired t-test.

## Results

### Suppression of psoriasin expression by IFN-gamma

**Figure 1:**
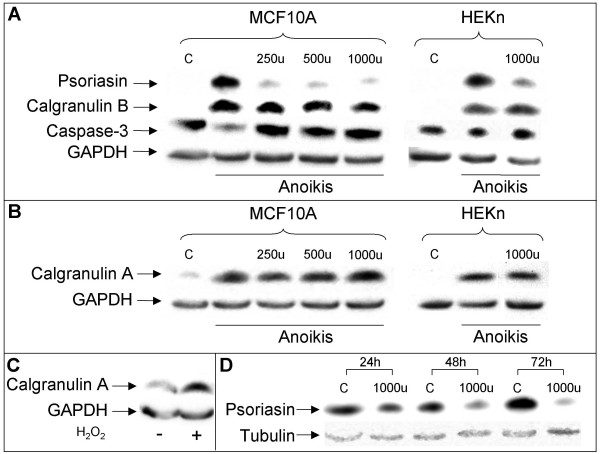
**Psoriasin expression is suppressed by IFN-gamma**. **A**, Suspension culture of MCF10A and HEKn shows the repression of psoriasin induction when treated 48 hours with 250 u, 500 u or 1000 u of IFN-gamma. IFN-gamma treatment had no effect on the protein levels of pro-caspase-3. Calgranulin B and calgranulin A (**B**) are not influenced by the IFN-gamma treatment. Equal amounts of protein lysate were loaded on the gel. **C**, Treatment with 75 μM H_2_O_2 _for one hour followed by culture for 48 h in regular medium induces the expression of calgranulin A in MCF10A cells. 100 μg of protein lysate were loaded on the gel. **D**, MDA-MB-468 cells with the endogenous expression of psoriasin show the time-dependent repression of psoriasin expression when treated 24, 48 and 72 hours with 1000 u of IFN-gamma. Equal amounts of protein lysate were loaded on the gel. Probing with tubulin/GAPDH assesses the equal loading of the samples. C = cells cultured in monolayer.

We tested the effect of IFN-gamma on the induction of psoriasin by anoikis (suspension culture), confluence and ROS, conditions which induce psoriasin and calgranulin B [7,20]. As depicted in Figure 1a, IFN-gamma treatment suppressed psoriasin induction by anoikis in MCF10A cells, as well as in HEKn cells, while it had no effect on psoriasin induction by ROS or confluent conditions (data not shown). We showed that, like psoriasin and calgranulin B, calgranulin A is also induced by these culture conditions (Figures 1b and 1c). However, we found no effect of IFN-gamma on calgranulin A and calgranulin B protein levels. In conclusion, we found that IFN-gamma treatment suppressed psoriasin induction by anoikis in two cell lines derived from two distinct epithelial tissues and that calgranulin A and calgranulin B were not modulated by the treatment (Figure 1a and 1b). IFN-gamma treatment had no effect on the protein levels of pro-caspase-3 (Figure 1a).

The ER-negative MDA-MB-468 breast carcinoma cell line has previously been shown constitutively to express psoriasin at high levels [5]. In accordance with the effect of IFN-gamma on psoriasin induction by anoikis, we showed that MDA-MB-468 cells treated with IFN-gamma also downregulate psoriasin (Figure 1d). We showed that MDA-MB-468 cells also display a high expression of calgranulin A and calgranulin B. However, similar to the findings in anoikis, the expression of calgranulin A and calgranulin B was not influenced by IFN-gamma treatment (data not shown).

### Downregulation of psoriasin expression occurs at the RNA level

**Figure 2:**
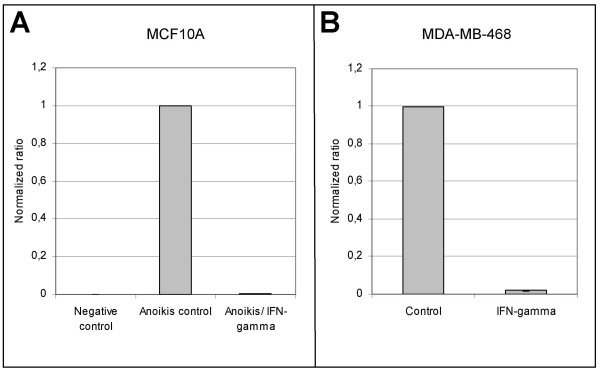
**Psoriasin mRNA levels are downregulated in response to IFN-gamma**. Cells were treated 48 hours with 350–1000 u of IFN-gamma and total RNA was prepared. Using real-time polymerase chain reaction (RT-PCR), mRNA level of psoriasin was downregulated in response to IFN-gamma in MCF10A cells (**A**) and in MDA-MB-468 cells (**B**). Expression data for psoriasin are presented as ratios, in which the expression data are normalized to an endogenous control (β-actin). Values obtained in control cells were designed as 1 and values obtained from IFN-gamma treated cells were normalized to this from the same run. The data are presented as the mean of three different runs.

The effect of IFN-gamma treatment was measured at the RNA level. Results of RT-PCR analyses (Figure 2) showed that the psoriasin RNA levels were dramatically reduced after treatment with IFN-gamma in both MDA-MB-468 and MCF10A cells. In fact, after 48 hours treatment with IFN-gamma the psoriasin expression level was reduced to only 0.5–2% of that of control cells. These data suggest that the effect of IFN-gamma on psoriasin expression is mediated at the transcriptional level.

### Activation of the STAT1 signaling pathway

**Figure 3:**
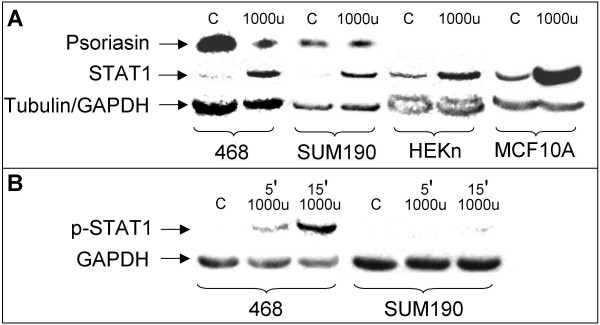
**IFN-gamma upregulates STAT1 expression and stimulates the phosphorylation of STAT1**. **A**, IFN-gamma upregulates STAT1 expression in MDA-MB-468, SUM190, HEKn, and MCF10A cells. The upregulation of STAT1 confirms the presence of the IFN-gamma receptor (48 hours' treatment with 1000 u of IFN-gamma). Endogenous psoriasin expression in MDA-MB-468 is downregulated by IFN-gamma treatment. However, in the SUM190 cell line, endogenous psoriasin expression was not affected by the treatment. Equal amounts of protein lysate for the different cell lines were loaded on the gel. Tubulin assesses equal loading in MDA-MB-468, HEKn and MCF10A cells. GAPDH assesses equal loading in SUM190 cells. **B**, Treatment with 1000 u of IFN-gamma led to the phosphorylation of STAT1 five and 15 minutes after IFN-gamma treatment in MDA-MB-468 cells, which confirms an active signaling pathway. Reduced phosphorylation of STAT1 was observed in the SUM190 cell line. 100 μg of protein lysate were loaded on the gel. GAPDH assesses equal loading. C control.

IFN-gamma is an important activator of STAT1 signaling [21]. To determine whether the downregulation of psoriasin involved the activation of STAT1, we studied the IFN-gamma/Jak/STAT1 pathway using antibodies against STAT1 and phosphorylated STAT1. The induction of STAT1 confirms the presence of the IFN-gamma receptor and the phosphorylated form confirms an active signaling pathway. In agreement with this, we demonstrated the upregulation of STAT1 in MDA-MB-468 cells, HEKn cells and MCF10A cells after 48 hours' treatment with IFN-gamma (Figure 3a). IFN-gamma treatment led to the phosphorylation of the STAT1 protein (Tyr-701) within a few minutes, thus confirming an active signaling pathway (Figure 3b).

The SUM190 breast carcinoma cell line also has high endogenous psoriasin protein levels. IFN-gamma did not downregulate psoriasin expression in this cell line, although the presence of the IFN-gamma receptor could be confirmed (Figure 3a). Interestingly, IFN-gamma treatment did not lead to a marked phosphorylation of the STAT1 protein, as we observed in the MDA-MB-468 cell line. This finding implies that STAT1 signaling is required for the IFN-gamma-mediated suppression of psoriasin expression (Figure 3b).

### Effect of IFN-gamma on cell viability and caspase activity

**Figure 4:**
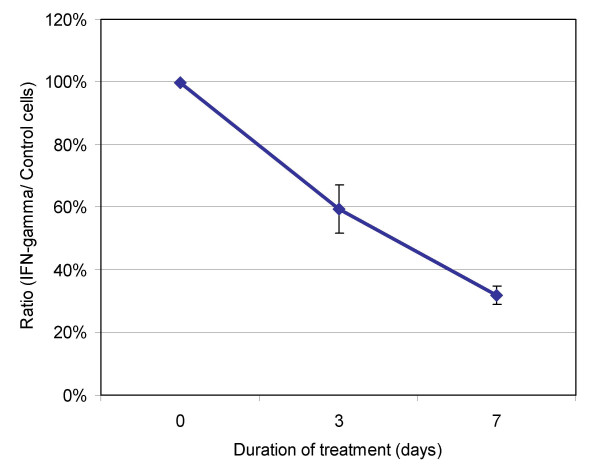
**IFN-gamma treatment inhibits the viability of mammary epithelial cells**. Mammary epithelial MCF10A cells were treated with 500 u of IFN-gamma at seeding. Cell viability was measured using the MTS-assay on days 3 and 7 and the IFN-gamma/control quotient is demonstrated. Treatment with IFN-gamma produced a 59.5% (day 3) and 32% (day 7) reduction in viability as compared to control cells. The assay was performed in duplicate.

In order to determine the effect IFN-gamma had on cellular viability, normal mammary epithelial MCF10A cells were incubated with 500 u of IFN-gamma. Using the MTS assay, we showed that cell viability was reduced by 32% after 7 days (Figure 4).

**Figure 5:**
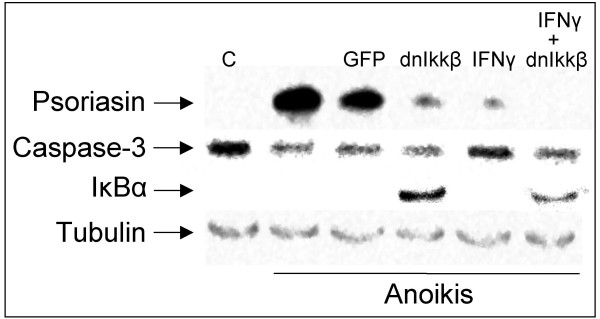
**IFN-gamma and the inhibition of NFκB suppress anoikis-induced psoriasin expression**. 500 u of IFN-gamma suppresses the psoriasin expression induced by anoikis for 48 hours either by itself or in combination with dnIkkβ-virus in MCF10A cells. Neither infection with the different adenoviruses nor the IFN-gamma had any effect on the protein levels of pro-caspase-3. The function of the virus was verified by the accumulation of the IκBα protein. Tubulin assesses equal loading. C control.

IFN-gamma treatment and the inhibition of NFκB were shown to have a synergistic effect on psoriasin levels (Figure 5). NFκB was blocked using a phosphorylation-defective dnIkkβ virus, which blocks the phosphorylation of IκBα necessary for NFκB activation. Figure 5 shows that dnIkkβ suppresses psoriasin induction and leads to the accumulation of the NFκB inhibitor IκBα. There was no difference in pro-caspase-3 level after dnIkkβ expression and IFN-gamma treatment, suggesting that the reduction in the induction of psoriasin following the expression of dnIkkβ and IFN-gamma is not due to increased apoptosis (Figure 5).

### Increased viability in psoriasin-expressing TAC2 cells

**Figure 6:**
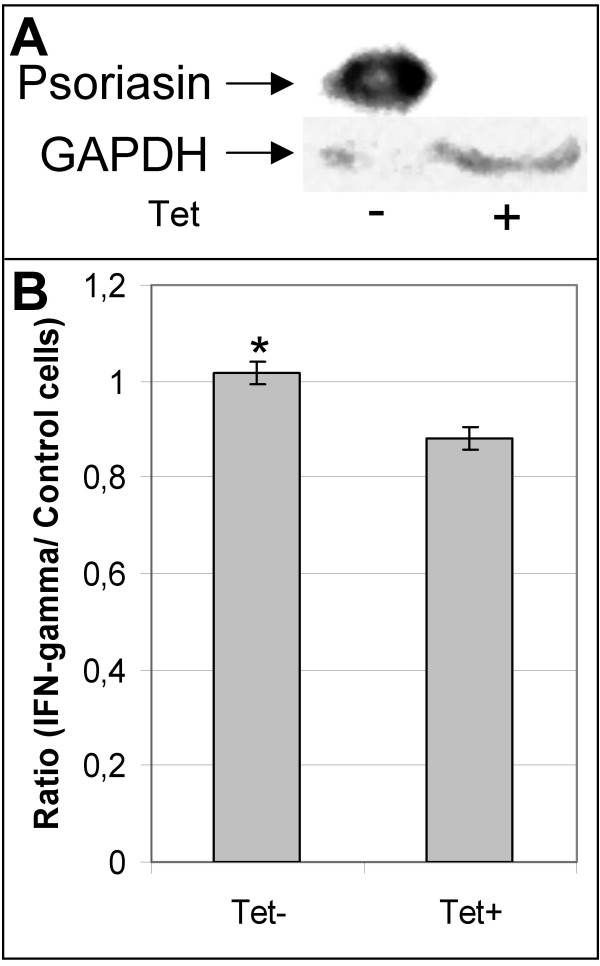
**Psoriasin reduces sensitivity to IFN-gamma- induced cell death**. **A**, Incubation with (+) and without (-) tetracycline in the tetracycline-responsive TAC2-tet EGFP2-psoriasin cell line demonstrates a significant amount of psoriasin expression in the absence of tetracycline. With tetracycline, the expression of psoriasin transcription is effectively suppressed. GAPDH assesses equal loading. 100 μg of protein lysate were loaded on the gel. **B**, Mammary epithelial TAC2 cells with tetracycline-inducible (Tet-Off) psoriasin expression (TAC2-tet EGFP2-psoriasin) demonstrate 15% higher viability in psoriasin-expressing cells (Tet-) than non-expressing (Tet+) when IFN-gamma (1000 u- 2000 u) is added at seeding (* p = 0.01, one-tailed paired t-test). Viability was measured by MTS assay on day 3 and the quotient IFN-gamma/control is demonstrated. The assay was performed in duplicate.

To study the functional relevance of high psoriasin expression in mammary epithelial cells, we established a TAC2 cell line expressing psoriasin in an inducible manner using the Tet-Off system. Several clones with low basal and high inducible psoriasin levels were isolated (Figure 6a). We found no significant difference between the proliferation of psoriasin-expressing TAC2 cells and control TAC2 cells (data not shown). We have previously shown an increase in the survival of psoriasin-expressing MCF10A cells in response to ROS, compared with control cells, and have demonstrated that this increase in survival is likely to be mediated by the NFκB pathway. Like ROS, IFN-gamma treatment leads to decreased viability in mammary epithelial cells (Figure 4). To determine whether psoriasin has an effect on cell viability after IFN-gamma treatment, we used mammary epithelial TAC2 cells with tetracycline-inducible (Tet-Off) psoriasin expression. Psoriasin-expressing TAC2 cells and control TAC2 cells were treated with IFN-gamma and viability was measured by MTS assay. The viability of the psoriasin-expressing TAC2 cells was 15% higher than in the non-expressing TAC2 cells when IFN-gamma was added at seeding (p = 0.01) (Figure 6b). Interestingly, psoriasin-expressing cells showed a resistant phenotype only when IFN-gamma was added when cells were plated. Treatment after cell attachment did not confer any difference in cell viability (data not shown).

## Discussion

IFN-gamma is a cytokine with diverse biological effects mediated by a variety of responsive genes. The anti-tumoral role for this cytokine involves both immunologic and non-immunologic processes. IFN-gamma activates cells in the innate immune system to proliferate and to produce cytokines [22]. In addition, IFN-gamma may have direct anti-proliferative and pro-apoptotic effects on a variety of tumor cells [16]. The activation of the interferon-stimulated genes (ISG) requires the activity of STAT1 transcription factor [21]. IFN-gamma is a potent activator of STAT1 and activated, phosphorylated STAT1 has been correlated to a good prognosis in breast cancer [23]. IFN-gamma inhibits the cell growth of mammary carcinoma cells and also reduces the angiogenic phenotype [17,18]. In metastatic breast cancer, IFN-gamma has been shown to enhance the growth-inhibitory effects of tamoxifen [24,25]. Moreover, a novel, promising anticancer drug, indole-3-carbinol (13C), was shown to increase the expression of the IFN-gamma receptor 1 and augmented the interferon responsiveness in breast cancer cells [26]. The characterization of genes that are regulated by IFN-gamma may therefore lead to a better understanding of the mechanisms of the anti-tumor action, as well as the improved utilization of IFN-gamma in the treatment of breast cancer.

Psoriasin, calgranulin A and calgranulin B are calcium-binding proteins with diverse biological functions. They have a high degree of structural and sequence homology and all three proteins, and psoriasin in particular, are highly expressed in DCIS and in the hyperproliferative skin disorder, psoriasis. We recently showed, by the hierarchical clustering of S100 gene expression in 22 breast cancer SAGE libraries, that one group with a distinguishable S100 gene expression profile was characterized by the high concomitant expression of psoriasin, calgranulin A and calgranulin B [27]. We are therefore focusing our interest not only on psoriasin but also on all three proteins.

We have now shown that psoriasin induction in MCF10A cells in anoikis (suspension culture) was suppressed after IFN-gamma exposure, at both the protein and mRNA level. The same effect was observed in the HEKn cell line, suggesting that this effect was not tissue specific. The MDA-MB-468 breast cancer cell line, which has endogenously produced psoriasin expression, also reduced the psoriasin expression level after IFN-gamma treatment. The cells treated with IFN-gamma displayed the upregulation and phosphorylation of STAT1, which indicates an active signaling pathway. These findings suggest that IFN-gamma mediates its suppressive effect on psoriasin transcription by activating the STAT1 signaling pathway. This conclusion is further supported by the reduced phosphorylation of the STAT1 protein after IFN-gamma treatment in the SUM190 cell line, where the high endogenous expression of psoriasin was not affected by the IFN-gamma treatment.

We have previously reported the increased survival of psoriasin-expressing cells after ROS treatment [7]. We now also show the increased viability in psoriasin-expressing mammary TAC2 cells after IFN-gamma treatment, which inhibits the cell growth of mammary carcinoma cells. High-grade comedo DCIS tumors demonstrate high apoptosis rates [28] and surviving tumor cells are also likely to be relatively more resistant to apoptosis. ROS thus lead to the endogenous production of psoriasin, which may contribute to apoptosis resistance in DCIS and psoriasis. The cytoprotective role of psoriasin in IFN-gamma-induced cell death is in line with the reported anti-apoptotic role of NFκB in several cell types, such as the protection from Tumor Necrosis Factor-α-induced apoptosis [29]. The increased viability seen in psoriasin-expressing cells could therefore be mediated through an NFκB-dependent pathway, which is supported by our data.

Interestingly, although all three S100 proteins are expressed in the MDA-MB-468 cancer cell line and in the normal epithelial cell lines in anoikis, IFN-gamma treatment had no effect on the expression levels of calgranulin A and calgranulin B. This may indicate that IFN-gamma interferes with signals that specifically modulate psoriasin expression.

In addition to the induction of psoriasin expression seen in anoikis, we have previously demonstrated the upregulation of psoriasin and calgranulin B in mammary epithelial cells (MCF10A) and keratinocytes (HEKn) in response to several other differentiation-promoting stimuli, such as an increase in extracellular calcium and confluent conditions [20]. We have also shown that psoriasin and calgranulin B are induced by ROS [7]. We now also show that calgranulin A is induced by ROS, which supports the assumption that these three S100 proteins share, at least in part, common signaling pathways in breast cancer. Our hypothesis is that, in all the conditions that induce psoriasin, calgranulin A and calgranulin B expression, ROS are produced. Accordingly, we have demonstrated the suppression of psoriasin and calgranulin B by the antioxidants Bcl-2 and N-acetyl-cysteine (NAC). However, we only observed the suppression of psoriasin by IFN-gamma in the anoikis condition, suggesting that the IFN-gamma-mediated signals implicated in psoriasin expression may only be present in anoikis and not in the other differentiation-promoting conditions.

We hypothesise that psoriasin is induced in anoikis by other stimuli and not only because of the presence of ROS. This would explain the massive induction seen in anoikis as compared with the other inducing conditions [3]. The finding that IFN-gamma suppresses psoriasin induction in anoikis lead to the hypothesis that the loss of adhesion signaling may contribute to the high psoriasin expression. Furthermore, the viability assay suggests that psoriasin expression may be related to the attachment of cells to the extracellular matrix; we observed that viability was higher in psoriasin-expressing cells when cells were treated with IFN-gamma at seeding compared with treatment following cell attachment. Attempts to identify the adhesion signals that interfere with psoriasin expression are currently ongoing.

The finding that psoriasin is downregulated by IFN-gamma has interesting clinical implications. IFN-gamma and STAT1 have previously been shown negatively to regulate angiogenesis. Recently, IFN-gamma was shown to suppress several of the angiogenic effects of VEGF [30]. We have previously shown that the downregulation of endogenous psoriasin in MDA-MB-468 cells led to the corresponding downregulation of VEGF and decreased tumour size [9]. The importance of psoriasin in mediating the anti-angiogenic properties of IFN-gamma remains to be determined.

## Conclusion

We report the suppression of psoriasin by IFN-gamma in two cell lines derived from two different normal epithelial tissues and in the ER-negative MDA-MB-468 breast carcinoma cell line. Our data implies that IFN-gamma mediates its suppressive effect on psoriasin transcription by activating the STAT1 signaling pathway. Psoriasin-expressing mammary epithelial cells showed increased viability after IFN-gamma exposure compared with normal cells.

## List of abbreviations

S100A7 = Psoriasin, S100A8 = Calgranulin A, S100A9 = Calgranulin B, Ductal carcinoma in situ = DCIS, Signal transducers and activators of transcription = STAT, Interferon-gamma = IFN-gamma, CellTiter 96^® ^AQ_ueous _One Solution Cell proliferation assay = MTS, Reactive oxygen species = ROS, Jun activating binding protein1 = Jab1, Vascular endothelial growth factor = VEGF, Poly-2-hydroxy-ethylmethacrylate = polyHEMA, Green Fluorescent Protein = GFP, Hydrogen peroxide = H_2_O_2_, Tet activator = tTA, and Crossing point = CP.

## Competing interests

The author(s) declare that they have no competing interests.

## Authors' contributions

SP and CE participated in designing the study, acquiring, analyzing and interpreting data, and preparing the manuscript. AB and MY contributed to acquisition and analysis of the data. All authors read and approved the final manuscript.

## Pre-publication history

The pre-publication history for this paper can be accessed here:


